# Safety and Short-term Efficacy of a Single Dose of 2 mg Moxidectin in *Loa loa*–Infected Individuals: A Double-Blind, Randomized Ivermectin-Controlled Trial With Ascending Microfilarial Densities

**DOI:** 10.1093/ofid/ofae240

**Published:** 2024-04-25

**Authors:** Guy S Wafeu, Tristan M Lepage, Jeremy T Campillo, Arnauld Efon-Ekangouo, Hugues-Clotaire Nana-Djeunga, Narcisse Nzune-Toche, André Domche, Laurentine Sumo, Guy-Roger Njitchouang, Martine Augusta Flore Tsasse, Jean Bopda, Yves Aubin Balog, Yannick Niamsi-Emalio, Stève Mbickmen-Tchana, Gervais Kamga Talla, Yannick Sédrick Nguedia Kana, Félicité Diane Maga Messina, Sébastien D Pion, Annette C Kuesel, Joseph Kamgno, Michel Boussinesq, Cedric B Chesnais

**Affiliations:** Epidemiology and Biostatistic Department, Higher Institute of Scientific and Medical Research, Yaoundé, Cameroon; TransVIHMI, Université de Montpellier, Inserm Unité 1175, Institut de Recherche pour le Développement, Montpellier, France; Department of Infectious and Tropical Diseases, Montpellier University Hospital, Montpellier, France; TransVIHMI, Université de Montpellier, Inserm Unité 1175, Institut de Recherche pour le Développement, Montpellier, France; Epidemiology and Biostatistic Department, Higher Institute of Scientific and Medical Research, Yaoundé, Cameroon; Epidemiology and Biostatistic Department, Higher Institute of Scientific and Medical Research, Yaoundé, Cameroon; Epidemiology and Biostatistic Department, Higher Institute of Scientific and Medical Research, Yaoundé, Cameroon; Epidemiology and Biostatistic Department, Higher Institute of Scientific and Medical Research, Yaoundé, Cameroon; Epidemiology and Biostatistic Department, Higher Institute of Scientific and Medical Research, Yaoundé, Cameroon; Department of Animal Biology and Physiology, University of Ebolowa, Ebolowa, Cameroon; Epidemiology and Biostatistic Department, Higher Institute of Scientific and Medical Research, Yaoundé, Cameroon; Epidemiology and Biostatistic Department, Higher Institute of Scientific and Medical Research, Yaoundé, Cameroon; Epidemiology and Biostatistic Department, Higher Institute of Scientific and Medical Research, Yaoundé, Cameroon; Epidemiology and Biostatistic Department, Higher Institute of Scientific and Medical Research, Yaoundé, Cameroon; Epidemiology and Biostatistic Department, Higher Institute of Scientific and Medical Research, Yaoundé, Cameroon; Epidemiology and Biostatistic Department, Higher Institute of Scientific and Medical Research, Yaoundé, Cameroon; Epidemiology and Biostatistic Department, Higher Institute of Scientific and Medical Research, Yaoundé, Cameroon; Epidemiology and Biostatistic Department, Higher Institute of Scientific and Medical Research, Yaoundé, Cameroon; Epidemiology and Biostatistic Department, Higher Institute of Scientific and Medical Research, Yaoundé, Cameroon; TransVIHMI, Université de Montpellier, Inserm Unité 1175, Institut de Recherche pour le Développement, Montpellier, France; Special Programme for Research and Training in Tropical Diseases, United Nations Children’s Fund/United Nations Development Programme/World Bank/World Health Organization, Geneva, Switzerland; Epidemiology and Biostatistic Department, Higher Institute of Scientific and Medical Research, Yaoundé, Cameroon; Department of Public Health, Faculty of Medicine and Biomedical Sciences, University of Yaoundé I, Yaoundé, Cameroon; TransVIHMI, Université de Montpellier, Inserm Unité 1175, Institut de Recherche pour le Développement, Montpellier, France; TransVIHMI, Université de Montpellier, Inserm Unité 1175, Institut de Recherche pour le Développement, Montpellier, France

**Keywords:** Africa, clinical trial, filariasis, loiasis, moxidectin

## Abstract

**Background:**

In 2018, the US Food and Drug Administration approved the macrocylic lactone moxidectin (MOX) at 8 mg dosage for onchocerciasis treatment in individuals aged ≥12 years. Severe adverse reactions have occurred after ivermectin (IVM), also a macrocyclic lactone, in individuals with high *Loa* microfilarial density (MFD). This study compared the safety and efficacy of a 2 mg MOX dose and the standard 150 µg/kg IVM dose in individuals with low *L loa* MFD.

**Methods:**

A double-blind, randomized, ivermectin-controlled trial of a 2 mg moxidectin dose was conducted in Cameroon between May and July 2022. It enrolled 72 adult men with *L loa* MFD between 5 and 1000 microfilariae/mL. Outcomes were occurrence of adverse events (AEs) and *L loa* MFD reduction rate during the first month off treatment.

**Results:**

No serious or severe AEs occurred among the 36 MOX- or the 36 IVM-treated individuals. Forty-nine AEs occurred in the MOX arm versus 59 AEs in the IVM arm. Grade 2 AE incidence was higher among IVM- than MOX-treated participants (38.5% and 14.3%, respectively, *P* = .043). Median MFD reduction rates were significantly higher after IVM than MOX at day 3 (70.2% vs 48.5%), day 7 (76.4% vs 50.0%), and day 30 (79.8% vs 48.1%).

**Conclusions:**

A single 2 mg MOX dose is as safe as 150 µg/kg IVM in patients with low *L loa* MFD. Further studies with higher MOX doses and in patients with higher MFD are warranted.

**Clinical Trials Registration:**

NCT04049851.

Onchocerciasis (“river blindness”) is a parasitic disease caused by the filarial nematode *Onchocerca volvulus*. Despite efforts to eliminate the disease, it remains a significant public health problem in sub-Saharan Africa. At least 244 million people in 30 countries require interventions to eliminate parasite transmission [1]. The World Health Organization (WHO) recommends community-directed treatment with ivermectin (CDTI) as a strategy, which has reduced morbidity and interrupted transmission in some areas. However, implementing CDTI in regions where loiasis, caused by the filarial parasite *Loa loa*, is also prevalent is challenging. Patients with high *L loa* microfilarial density (MFD) may experience serious adverse events (SAEs) after ivermectin (IVM) treatment [2, 3]. In addition, in areas where onchocerciasis is hypoendemic and loiasis coendemic, the risk of SAEs in individuals exceeds the benefit of treatment for the community, and CDTI is not implemented. This hampers the achievement of the elimination goal, and alternative treatment strategies, including the use of safe and more efficacious drugs, must be implemented in such areas [4].

Moxidectin (MOX), approved in 2018 by the US Food and Drug Administration for onchocerciasis treatment of individuals aged ≥12 years, shows promise for achieving elimination goals [5]. It has a stronger and longer-lasting effect on *O volvulus* microfilaridermia compared to IVM [6–8]. Since MOX is structurally similar to IVM [9], and as both drugs have a microfilaricidal effect on *O volvulus*, MOX may result in similar adverse events (AEs) in *L loa*–infected patients. This study aims to evaluate for the first time the safety of MOX (given at low dose of 2 mg) in individuals with low *L loa* MFD and to compare the kinetics of *L loa* MFD after a single dose of MOX or IVM.

## METHODS

### Study Design and Participants

In this double-blind, randomized controlled trial, a single 2 mg oral dose of MOX (experimental arm; see Supplementary Material 1 for the justification of the 2 mg MOX dose) was compared to a standard oral dose of IVM (150 µg/kg) (control arm). The first group (cohort 1) included individuals with an MFD <100 *L loa* microfilariae (mf)/mL blood to minimize the risk of AEs. If no SAEs occurred within 72 hours of treatment (day 3 [D3]) in cohort 1, individuals with up to 500 mf/mL were treated (cohort 2). Cohort 3, consisting of individuals with up to 1000 mf/mL, was treated after no SAEs were observed up to D3 in cohort 2. These low MFD values were selected assuming a minimal risk of SAEs even if MOX had a stronger microfilaricidal effect on *L loa* mf than IVM.

The trial was conducted in 2 rural health districts (Mbalmayo and Awae) in the Centre region of Cameroon, located approximately 30 km from the capital, Yaoundé. Loiasis is prevalent with microfilaremia rates exceeding 30% in some villages of these districts.

### Randomization and Blinding

Visually identical capsules containing a 2 mg MOX tablet, a 3 mg IVM tablet, or a placebo (no tablet) were manufactured by Almac Sciences (Ireland) Ltd. Randomization lists (block size 3) were generated by an independent statistician (Y. N.-E.) for each cohort, with a 1:1 allocation ratio to either 2 mg MOX or 150 µg/kg IVM treatment. Stratification was based on age (below or above the cohort’s median age). An independent pharmacist allocated randomization codes and prepared sealed envelopes with the capsules, following the age and cohort appropriate randomization lists. Participants, investigators, and the data analyst were blinded. The capsules (total of 5 for each participant) were administered under direct observation by a physician.

### Sample Size

As no data were available on the safety or efficacy of moxidectin on *L loa* MFD, the sample size was only calculated based on the frequency of clinical AEs expected to occur within 7 days of drug administration. In a previous study conducted in a *L loa*–endemic area, in which >15 000 participants had been monitored for AEs occurring after a single dose of IVM (150 µg/kg IVM) [10], the incidence of AEs during the first 7 days after treatment in individuals with ≤1000 mf/mL was 3.2% (no cases of SAEs were observed). Compared to clinical trials registration where 80 individuals by arm were planned to provide a 90% probability of detecting at least 1 AE with a true frequency of 3%, a first Cameroonian ethical amendment aimed at including 55 individuals in each arm, providing an 80% probability of detecting at least 1 AE with a true frequency of 3%.

### Eligibility Criteria

Inclusion criteria for the trial were (1) males aged 18–70 years with a body weight between 45 and 85 kg; (2) normal hematology parameters defined as leukocyte count between 2800 and 11 300 cells/μL, hemoglobin ≥10 g/dL, and platelet count ≥100 000/μL; (3) creatininemia ≤1.3 mg/dL (≤2.5 upper normal limit [UNL]); (4) total bilirubinemia ≤3.3 mg/dL (≤2.5 UNL); (5) γ-glutamyltransferase ≤183 IU/L (≤3 UNL); (6) serum alanine aminotransferase level ≤113 IU/L (≤2.5 UNL); (7) normal urinalysis values; and (8) *L loa* MFD between 5 and 1000 mf/mL. Exclusion criteria were treatment with IVM during the previous 6 months, ongoing antiretroviral therapy or treatment with ampicillin or chloramphenicol during the last 10 days, any acute infection during the last 10 days before treatment, known allergy to IVM or MOX, and any other condition/comorbidity which, in the investigators’ opinion, would expose the individual to undue risk.

### Procedures

A survey was conducted in February–March 2022 to identify males aged 18–70 years potentially meeting the *L loa* MFD eligibility criterion. Fifty microliters of blood was collected by finger prick between 10:00 Am and 4:00 Pm to account for the diurnal periodicity of *L loa* microfilaremia. One thick blood smear (TBS) was prepared, dried, dehemoglobinized, stained with Giemsa, and examined using a microscope to assess the *L loa* MFD. Individuals with an MFD between 10 and 1500 mf/mL were further evaluated few weeks ago with clinical examinations, urinalysis, venous blood analysis, and 2 new TBSs. The upper limit of 1500 mf/mL, allowing the inclusion of individuals with a maximum of 1000 mf/mL 3 weeks after the initial survey, was determined based on our field experience, acknowledging the variability of *L loa* MFD over time [11]. Independent laboratory technicians (J. B., S. M.-T.) read each of the 2 slides twice, and the arithmetic mean of the 4 readings was considered for analysis (the minimum MFD possible was 5 mf/mL, when only 1 mf was found at 1 reading).

Cohorts 1, 2, and 3 were treated from 25 May, 26 June, and 20 July 2022, respectively. Follow-up visits occurred on day 3 (D3), day 7 (D7), and day 30 (D30) after treatment. Each follow-up visit included a clinical examination, a questioning for AEs, and 2 additional TBS slides. On D7, a 10 mL venous blood sample was collected to assess changes in hematology or serum biochemistry parameters and determine if they met laboratory AE criteria. Each of the clinical manifestations during follow-up was considered as a distinct AE if no syndromic diagnosis was present. In the event of AEs, patients had the possibility to call the team for assistance and receive appropriate care (a phone number was provided on the participant's card). In the case of clinical AEs, data from missed visits were diligently collected during subsequent visits or through phone calls to ensure a comprehensive and accurate reporting of events.

### Outcomes

The primary outcome was treatment safety, assessed by the occurrence of possibly drug-related SAEs as well as the incidence and severity of any potentially drug-related AEs from day zero (D0) to D7 posttreatment for laboratory AEs and from D0 to D30 for clinical AEs. Clinical and laboratory AEs were graded according to the Common Terminology Criteria for Adverse Events version 5.0, where SAEs are defined from grade 3 onward, with grade 5 AEs representing cases of death. The change in laboratory parameters for each participant was quantified as follows: 100 × [(D7 value) – (baseline value)] / [baseline value]. Serum creatinine variation between baseline and D7 was categorized based on Kidney Disease—Improving Global Outcomes (KDIGO) guidelines, defining grade 1 acute kidney injury as a 1.5- to 1.9-fold increase from baseline or a rise above 0.3 mg/dL, and grade 2 as an elevation of 2.0 to 2.9 times the baseline value [12].

The secondary outcome, efficacy, was quantified in 2 ways: (1) the *L loa* MFD reduction rates from baseline to D3, D7, and D30, calculated as 100 × [(baseline MFD) – (MFD at D_3, 7, and 30_)] / [baseline MFD]; and (2) the proportion of individuals with MFD reduction rates of >40%, >80%, and 100% (microfilaria clearance: *L loa* MFD reduction to 0 mf/mL).

### Statistical Analysis

Descriptive parameters presented by treatment arm are counts and percentages for categorical variables, means and standard deviations for normally distributed quantitative data, and medians (interquartile ranges) or geometric means (95% confidence intervals) for skewed variables. Categorical variables were compared using Pearson χ^2^ or Fisher exact test as appropriate. Descriptive bivariate statistics tables were generated comparing AE incidence by participants’ age, initial *L loa* MFD, and the presence of *Mansonella perstans* (another filarial species endemic in the study area) mf in the TBS.

*Loa* MFD reduction rates (from D0 to D3, D7, or D30) and laboratory parameter changes (from D0 to D7) between treatment arms were compared using nonparametric Wilcoxon rank-sum tests.

Data were analyzed with IBM SPSS Statistics for Windows, version 26.0 (IBM, Armonk, New York) and with R-Cran statistical environment, version 4.2.0. The main data analysis was performed using an intent-to-treat approach. In addition, a sensitivity analysis of efficacy was performed using a per protocol approach. We conducted a pooled analysis of the 3 cohorts to circumvent low-powered statistical tests and minimize the potential consequences of multiple testing.

### Trial Registration

This study was conducted in accordance with the rules of Good Clinical Practice. The trial was registered on ClinicalTrials.gov (NCT04049851).

## RESULTS

### Screening and Eligibility

**Figure 1.**
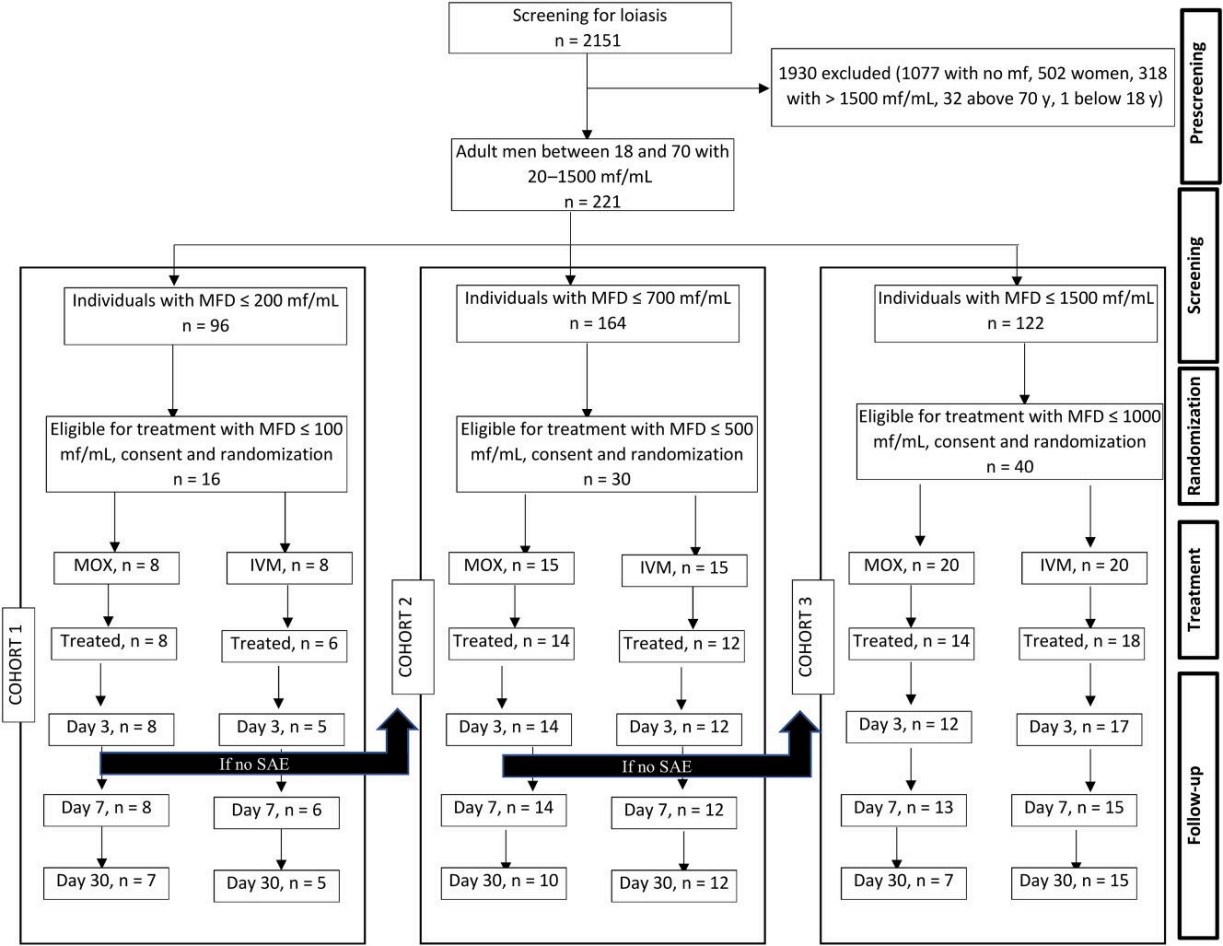
Flowchart of participants screened, randomized, and included in the trial for each cohort and arm. Abbreviations: IVM, ivermectin; mf, microfilariae; MFD, microfilarial density; MOX, moxidectin; SAE, serious adverse event.

From February to March 2022, 2151 males were screened for *L loa* MFD with a single TBS. Among them, 221 had an MFD between 20 and 1500 mf/mL and underwent further eligibility screening, including reading of 2 new TBSs by 2 microscopists. A total of 86 participants were randomized, with 72 receiving treatment (14 for cohort 1, 26 for cohort 2, and 32 for cohort 3). Nine participants had developed an acute infection before treatment, and others withdrew consent (Supplementary Material 2). Day 30 evaluation was performed for 24 participants in the MOX arm and 32 in the IVM arm. Additional details are presented in Figure 1.

### Baseline Characteristics

Participants’ mean age was 51.5 years in the MOX arm and 51.9 years in the IVM arm. Baseline characteristics were similar between arms. The median *L loa* MFD in the MOX and IVM arms was 197.5 and 282.5 mf/mL, respectively (Table 1). *Mansonella perstans* mf were found in the blood of 22.2% and 25.0% of participants in the MOX and IVM arms, respectively.

**Table 1.** Baseline (Pretreatment) Characteristics of Participants

Variable	Cohort 1		Cohort 2		Cohort 3		Overall	
	MOX (n = 8)	IVM (n = 6)	MOX (n = 14)	IVM (n = 12)	MOX (n = 14)	IVM (n = 18)	MOX (n = 36)	IVM (n = 36)
Age, y^a^	54.6 ± 14.3	45.2 ± 10.6	47.5 ± 16.8	56.7 ± 8.5	53.8 ± 14.7	50.9 ± 12.8	51.5 ± 15.4	51.9 ± 11.6
*Loa loa* MFD								
Median (IQR)	27.5 (15–42.5)	37.5 (30–45)	267.5 (185–370)	282.5 (162.5–350.0)	337.5 (45–530)	460 (185–770)	197.5 (35–407.5)	282.5 (91.3–471.3)
Min–Max	5–60	10–90	10–465	45–475	10–925	10–980	5–925	10–980
GM (95% CI)	22.8 (13.2–38.2)	34.6 (20–55.9)	195.7 (108.8–304.6)	227.3 (150.6–324.7)	166.9 (74–344.9)	324.5 (191.4–504.9)	114.1 (69.2–184.0)	198.4 (132.5–295.0)
*Mansonella perstans*								
Prevalence, No. (%)	3 (37.5)	2 (33.3)	1 (7.1)	3 (25.0)	4 (28.6)	4 (22.2)	8 (22.2)	9 (25)
Median (IQR)	0 (0–225)	0 (0–25)	0 (0–0)	0 (0–32.5)	0 (0–10)	0 (0–0)	0 (0–0)	0 (0–18.8)
Min–Max	0–3265	0–75	0–45	0–2765	0–2995	0–135	0–3265	0–2765
Heart rate^a^	72.1 ± 9.9	81.5 ± 14.0	71.8 ± 14.5	74.2 ± 15.5	71.0 ± 10.7	67.8 ± 10.0	71.6 ± 11.9	69.9 ± 12.4
Mean BP^a^	94.1 ± 10.1	95.4 ± 16.4	94.3 ± 18.3	100.2 ± 16.1	93.8 ± 10	93.3 ± 12	94.0 ± 13.4	95.9 ± 14.1
Systolic BP^a^	131.9 ± 19.5	123.3 ± 21.6	125.0 ± 22.7	130.8 ± 22.8	127.1 ± 14.9	122.9 ± 13.7	127.4 ± 18.9	125.6 ± 18.3
Diastolic BP^a^	75.3 ± 9.3	81.5 ± 14.0	78.9 ± 16.9	84.8 ± 14.6	77.1 ± 10.8	78.5 ± 11.9	77.4 ± 13.0	81.1 ± 13.1
Temperature^a^	36.0 ± 0.8	36.5 ± 0.5	36.1 ± 0.5	35.9 ± 1.2	36.3 ± 0.5	36.2 ± 0.6	36.2 ± 0.6	36.1 ± 0.8

Abbreviations: BP, blood pressure; CI, confidence interval; GM, geometric mean; IQR, interquartile range; IVM, ivermectin; MFD, microfilarial density (in number of microfilariae/mL of blood); MOX, moxidectin; n, number of subjects with *Mansonella perstans* microfilariae.

^a^Data are mean ± standard deviation.

### Safety Data

Eight AEs were unrelated to the intervention: 3 malaria episodes, 1 malaria-associated hyperbilirubinemia, 2 traumatic injuries, 1 antibiotic-associated diarrhea, and 1 otitis media. All other AEs were considered possibly drug-related and included in the analysis. In the MOX arm, 49 AEs occurred: 31 clinical AEs (from D0 to D30) and 18 laboratory AEs (from D0 to D7) (Table 2). In the IVM arm, 59 AEs were recorded within the same time frame (43 clinical and 16 laboratory AEs). Median onset times of AEs did not significantly differ between arms (Table 2). There were no significant differences in the percentage of participants with at least 1 AE between the MOX and IVM arms (77.8% vs 72.2%, *P* = .586), whether in case of clinical (55.6% vs 63.9%, *P* = .471) or laboratory AEs (42.9% vs 33.3%, *P* = .419). No SAE or grade 3 or 4 AE occurred. In the MOX arm, 85.7% of subjects with at least 1 AE had grade 1 severity, compared to 61.5% in the IVM arm. Grade 2 AEs were significantly more frequent in the IVM arm (Table 2). There were no significant differences in the type of clinical (Table 3) or laboratory (Table 4) AEs between arms. The most frequent clinical AEs were headache, rhinorrhea, fatigue, fever, and pruritus. The most frequent laboratory abnormality was grade 1 creatinine increase, recorded in 25.7% and 18.2% of participants in the MOX and IVM arms, respectively. Neutropenia occurred in 8.6% of the participants of the MOX arm (all grade 1) and 18.2% in the IVM arm (including 1 grade 2 neutropenia, *P* = .299). No other notable abnormalities occurred. Details about clinical (stratified by age, by *L loa* MFD and per protocol analysis) and laboratory AEs are provided in the Supplementary Materials 3–6 and 11. Last, there were no significant difference in AE frequency according to the presence or absence of *M perstans* microfilariae (Supplementary Material 7).

**Table 2.** Number of Adverse Events (AEs) Possibly Related to Treatment, Number of Subjects Having Developed Such AEs, and Interval of Time Between Treatment and Onset of AEs in the 2 Treatment Arms

Adverse Events	MOX (n = 36)^a^	IVM (n = 36)^a^	*P* Value
Median onset, d (IQR)	2.5 (1.0–6.8)	2.0 (1.0–5.2)	.794^b^
Mean onset, d (IQR)	5.3 (1.0–6.8)	4.2 (1.0–5.2)	.794^b^
No. of AEs	49	59	
Clinical AEs	31	43	
≤7 d	25	35	
>7 d^c^	6	8	
Laboratory AEs at day 7	18	16	
No. (%) of subjects with AEs			
Any AEs	28 (77.8)	26 (72.2)	.586^d^
Clinical AEs	20 (55.6)	23 (63.9)	.471^d^
≤7 d	16 (44.4)	21 (58.3)	.238^d^
>7 d^c^	6 (16.7)	4 (11.1)	.496^d^
Laboratory AEs^e^	15 (42.9)	11 (33.3)	.419^d^
Maximum grade reached			
Grade 1 AE^f^	24 (85.7)	16 (61.5)	.043^g^
Grade 2 AE^f^	4 (14.3)	10 (38.5)	
Grade 3/4 AE	0 (0)	0 (0)	

Abbreviations: AE, adverse event; IQR, interquartile range; IVM, ivermectin; MOX, moxidectin.

^a^n: Number of participants included in the arm.

^b^Wilcoxon rank-sum test.

^c^Clinical AEs were reported up to 30 d.

^d^Pearson χ^2^ test.

^e^The proportions of laboratory AEs are calculated on those individuals who attended the day 7 visit (n = 35 for MOX and n = 33 for IVM).

^f^In these lines, the proportions are calculated only on those individuals who have developed an AE.

^g^Fisher exact test.

**Table 3.** Number of Subjects Having Reported Each Type of Clinical Adverse Event Possibly Related to Moxidectin or Ivermectin Treatment Within 30 Days After Treatment, by Treatment Arm

Adverse Event	MOX (n = 36)	IVM (n = 36)	*P* Value
Gastrointestinal disorders	5 (13.8)	5 (13.8)	.999^a^
Diarrhea	2 (5.6)	1 (2.8)	
Constipation	2 (5.6)	0 (0)	
Epigastric pain	0 (0)	3 (8.3)	
Abdominal pain	1 (2.8)	0 (0)	
Jaundice	0 (0)	1 (2.8)	
Gastrointestinal bleeding	1 (2.8)	0 (0)	
Nervous system disorders	5 (13.8)	4 (11.1)	.999^b^
Headache	4 (11.1)	4 (11.1)	
Vertigo	1 (2.8)	0 (0)	
Eye disorders	3 (8.3)	2 (5.6)	.999^b^
Watering eyes	1 (2.8)	0 (0)	
Ocular pruritus	2 (5.6)	2 (5.6)	
Respiratory disorders	0 (0)	5 (13.8)	.054^b^
Rhinorrhea	0 (0)	5 (13.8)	
Cough	0 (0)	1 (2.8)	
Musculoskeletal disorders	3 (8.3)	6 (16.7)	.478^b^
Arthralgia	3 (8.3)	3 (8.3)	
Low back pain	0 (0)	3 (8.3)	
General disorders	6 (16.7)	8 (22.2)	.551^a^
Fatigue	4 (11.1)	6 (16.7)	
Fever	3 (8.3)	6 (16.7)	
Skin disorders	7 (19.4)	7 (19.4)	.999^a^
Pruritus	4 (11.1)	5 (13.8)	
Soft tissue edema	2 (5.6)	2 (5.6)	
Maculopapular rash	1 (2.8)	0 (0)	
Cardiovascular disorders	0 (0)	1 (2.8)	.999^b^
Hypertension	0 (0)	1 (2.8)	

Data are presented as No. (%) unless otherwise indicated.

Abbreviations: IVM, ivermectin; MOX, moxidectin.

^a^Pearson χ^2^ test.

^b^Fisher exact test.

**Table 4.** Number of Subjects With Laboratory Adverse Events Possibly Related to Moxidectin or Ivermectin Treatment Between Baseline and Day 7, by Treatment Arm

Adverse Events	MOX (n = 35)	IVM (n = 33)	*P* Value
Blood parameters			
Creatinine increased	9 (25.7)	6 (18.2)	.454^a^
ALT increased	0 (0)	0 (0)	.999^b^
GGT increased	1 (2.9)	1 (3.0)	.999^b^
Bilirubin increased	3 (8.6)	2 (6.1)	.999^b^
Anemia	1 (2.9)	2 (6.1)	.999^b^
Leukopenia	1 (2.9)	1 (3.0)	.999^b^
Neutropenia	3 (8.6)	6 (18.2)	.299^b^
Lymphopenia	0 (0)	0 (0)	.999^b^
Thrombocytopenia	0 (0)	0 (0)	.999^b^
Urine dipstick			
Leukocyturia	0 (0)	0 (0)	.999^b^
Proteinuria	0 (0)	0 (0)	.999^b^
Hematuria	0 (0)	0 (0)	.999^b^

Data are presented as No. (%) unless otherwise indicated.

Abbreviations: ALT, alanine aminotransferase; GGT, γ-glutamyltransferase; IVM, ivermectin; MOX, moxidectin.

^a^Pearson χ^2^ test.

^b^Fisher exact test.

### Effect of *L loa* MFD

**Figure 2.**
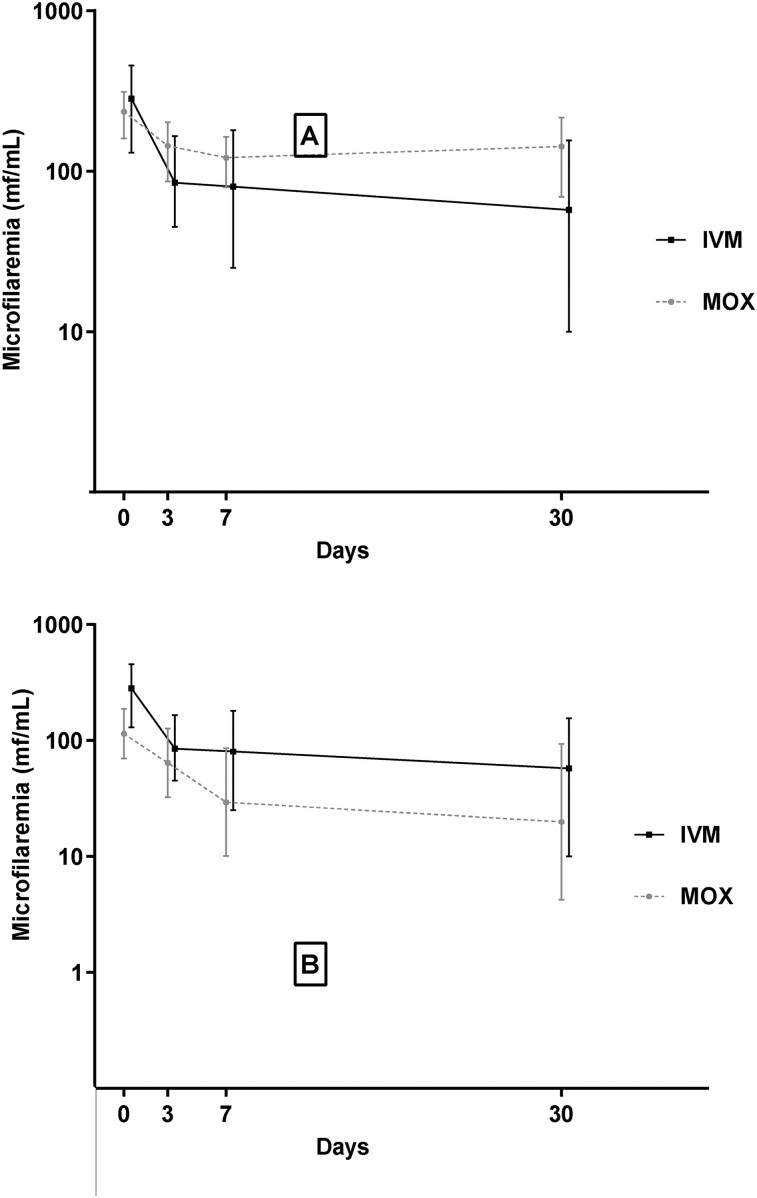
Evolution of microfilaremia at days 3, 7, and 30, showing median with interquartile range (*A*), and geometric mean with 95% confidence interval (*B*). Abbreviations: IVM, ivermectin; mf, microfilariae; MOX, moxidectin.

**Figure 3.**
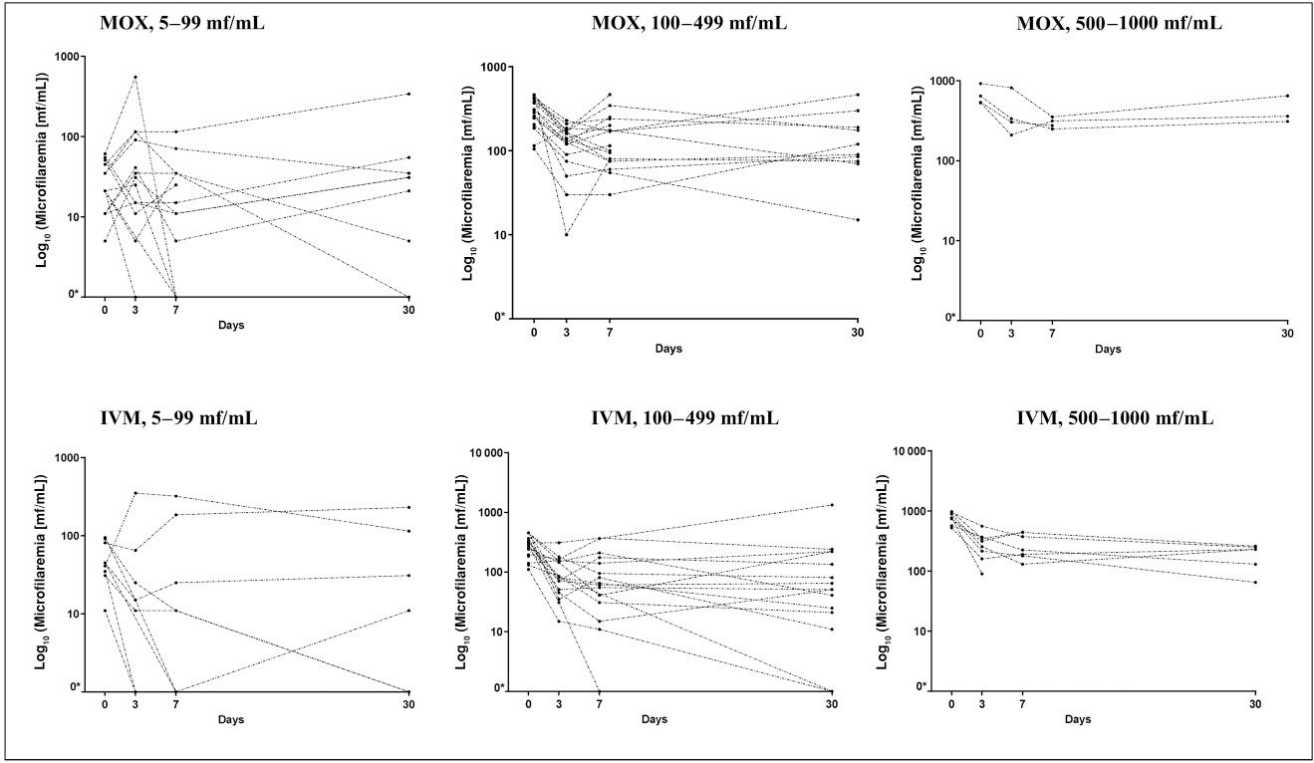
Microfilaremia of each individual treated by baseline microfilarial density (MFD). *To show all of the data on the graphs, all zero MFDs were considered as 1 and their log_10_ was zero. Abbreviations: IVM, ivermectin; mf, microfilariae; MOX, moxidectin.

At D3, the reduction in *L loa* MFD was significantly higher in the IVM arm compared to the MOX arm, with median reduction rates of 70.2% and 48.5%, respectively (*P* = .004). Similar significant differences between treatment arms were observed at D7 and D30, with median reduction rates of 76.4% and 79.8% in the IVM arm, and 50.0% and 48.1% in the MOX arm (Table 5). *Loa loa* MFD decreased between D0 and D7 and remained relatively stable from D7 to D30 in both arms (Figure 2). There was no significant effect on *M perstans* MFD (Supplementary Material 8). The proportion of participants with a >40% decrease in *L loa* MFD from baseline was not significantly different between the arms at D3, D7, and D30 (*P* = .272, *P* = .082, and *P* = .103, respectively). At D3 and D7, the proportion of participants with a reduction >80% of the initial *L loa* MFD was significantly higher in the IVM arm than in the MOX arm: 32.4% versus 11.8% at D3 (*P* = .041), and 39.4% versus 17.1% at D7 (*P* = .041). Microfilaraemia clearance at D3, D7, and D30 did not significantly differ between the treatment arms (Table 6). Patterns of individual *L loa* MFD changes are shown in Figure 3 for each baseline MFD category. Last, the per protocol analyses found similar results (Supplementary Materials 9 and 10).

**Table 5.** Mean *Loa loa* Microfilarial Density at Each Time Point and Mean and Median Individual Reduction Rates Between Baseline (Day 0) and Day 3, Day 7, and Day 30

Day	MOX		IVM		*P* Value^a^
	Median MFD (IQR)	Median Relative Difference, %	Median MFD (IQR)	Median Relative Difference, %	
Day 0	197.5 (35.0–392.5)	…	282.5 (93.8–463.8)	…	…
Day 3	112.5 (31.3–173.8)	48.5 (−52.7 to 64.7)	85.0 (31.3–180.0)	70.2 (42.6–85.0)	.004
Day 7	75.0 (27.5–187.5)	50.0 (−2.0 to 75.4)	80.0 (10.0–19.0)	76.4 (55.6–89.4)	.017
Day 30	72.5 (18.8–217.5)	48.1 (−12.7 to 81.7)	57.5 (7.5–220.0)	79.8 (39.0–99.5)	.047

Median MFDs are expressed in number of microfilariae/mL of blood.

Abbreviations: IQR, interquartile range; IVM, ivermectin; MFD, microfilarial density; MOX, moxidectin.

^a^Wilcoxon rank-sum test.

**Table 6.** Proportion of Participants With 40%, 80%, and 100% Reduction From Pretreatment in Their *Loa loa* Microfilarial Density

Day	40% Decrease					80% Decrease					Microfilarial Clearance				
	MOX		IVM		*P* Value	MOX		IVM		*P* Value	MOX		IVM		*P* Value
	Yes	No	Yes	No		Yes	No	Yes	No		Yes	No	Yes	No	
Day 3	23 (67.6)	11 (32.4)	27 (79.4)	7 (20.6)	.272	4 (11.8)	30 (88.2)	11 (32.4)	15 (22.1)	.041	1 (2.9)	33 (97.1)	3 (8.8)	31 (91.2)	.614
Day 7	22 (62.9)	13 (37.1)	27 (81.8)	6 (18.2)	.082	6 (17.1)	29 (82.9)	13 (39.4)	20 (60.6)	.041	4 (11.4)	31 (88.6)	6 (18.2)	27 (81.8)	.507
Day 30	13 (54.2)	11 (45.8)	24 (75)	8 (25)	.103	6 (25)	18 (75)	16 (50)	16 (50)	.058	4 (16.7)	20 (83.3)	8 (25)	24 (75)	.452

Data are presented as No. (%) unless otherwise indicated.

Abbreviations: IVM, ivermectin; MFD, microfilarial density; MOX, moxidectin.

## DISCUSSION

This trial is the first to assess the safety and efficacy of MOX in *L loa* microfilaremic individuals. The objective of this pilot clinical trial was to evaluate whether MOX could be used for onchocerciasis elimination in loiasis-endemic areas. The main obstacle in these areas being the risk of posttreatment SAEs in individuals with high *L loa* MFD, we chose to administer a low dose of MOX in patients with low *L loa* MFD for safety purposes. No SAEs or grade 3 or 4 AEs were recorded in either treatment arm, and we observed a significantly slower *L loa* MFD decrease in the MOX arm compared to the IVM arm. This will allow the development of clinical trials with higher *L loa* MFD and/or higher MOX doses.

Based on our previous study [10], we expected around 3.2% of IVM-treated subjects to have at least 1 clinical AE within 7 days. However, in this study, the proportion of individuals with at least 1 clinical AE after IVM treatment was substantially higher (58.3%). This difference may be due to 2 factors. First, active follow-up in this clinical trial increased the probability of recording low-grade AEs. In contrast, our previous community study relied on passive reporting, which may have missed such AEs. Second, participants in this study were more aware of potential AEs due to this new drug and reported even minor changes. These explanations are consistent with the lower proportion of grade 2 AEs in this trial compared to our previous study.

In our trial, 77.8% of participants treated with MOX had at least 1 possibly related AE within the 30 days posttreatment. This is lower than reported frequencies in trials evaluating MOX in *O volvulus–*infected individuals (86%–100% in different MOX treatment arms and 96%–97% in the 150 µg/kg IVM comparator arms) [6, 7]. A comparison of pruritus incidence shows lower rates in our trial for both MOX (11.1%) and IVM (13.8%) compared to *O volvulus*–infected individuals. Based on this comparison and the similar overall AE incidence after 2 mg, 4 mg, or 8 mg MOX treatment of *O volvulus*–infected individuals [6], the 2 mg dose of MOX in our study might not be the cause of the difference in AE incidence in *O volvulus* compared to *L loa*–infected individuals.

The fact that *O volvulus* mf are located in the dermis, and not in the blood as those of *L loa*, may contribute to the difference in post-MOX AE incidence between onchocerciasis and loiasis subjects. After IVM treatment, the motility of *O volvulus* mf in the skin tissues is reduced and the parasites are then attacked by adhering immunocompetent cells and drained within the lymphatic vessels up to the lymph nodes, where they are destroyed [13, 14]. This process induces Mazzotti reactions, including pruritus and edema. The phenomena occurring after MOX treatment in subjects with *O volvulus* mf have not been investigated but are probably similar to those documented after IVM treatment.

In the IVM arm, the median *L loa* MFD reduction rates at D3, D7, and D30 were 70.2%, 76.4%, and 79.8%, respectively, consistent with previously described MFD reduction kinetics [15]. The decrease in *L loa* MFDs during the first week of treatment was significantly faster in the IVM arm than in the MOX arm, as confirmed in the per protocol analysis (Supplementary Material 9). The lower effect of MOX on *L loa* mf may also explain the lower frequency of grade 2 AEs in the MOX arm, as AEs are mostly related to the microfilaricidal effect. Last, a relationship between *L loa* MFD and AE incidence (which is already well documented [16, 17]) was observed in both arms, although not significant in the MOX arm (Supplementary Material 4). Since the sample size plan was not designed to detect a significant difference in efficacy between our 2 groups, and in light of this somewhat unexpected outcome, this post hoc result requires cautious interpretation and underscores the need for future clinical trials with sampling plans and, especially, adapted sample sizes, to confirm or disprove this trend.

Due to logistic difficulties (mainly because of the coronavirus disease 2019 pandemic), the populations included in the screening phase were more hesitant than expected to participate in research studies. This situation led us to obtain a Cameroonian ethical amendment to reduce our sample size (see Methods). Despite this modification, field conditions led us to stop recruitment before reaching the targeted sample size, which was an important limitation to our study. However, as our hypotheses were based solely on the ability to detect at least 1 AE in each arm, the impact on safety and efficacy comparing results between our 2 groups cannot be measured. Regarding the occurrence of AEs, it appears that the reality exceeds what we could have expected in terms of our capacity to detect at least 1 AE, since the proportion of AEs is much higher than the 3% used in our sample size calculation. In addition, we observed an interindividual variability in microfilariae kinetics (which may be due to our small sample size), especially in the MOX arm (as illustrated with the positive mean reduction rates in Table 5, which may be mainly explained by the lowest stratum of *L loa* MFD, Figure 3). The latter should reinforce the importance of further studies to confirm the effect of MOX on *L loa* mf and understand its mechanism. A dose finding of MOX may also be envisioned in further trials.

In conclusion, the safety profile of 2 mg MOX in patients with low *L loa* MFDs was similar to that of IVM. A single 2 mg MOX dose reduced *L loa* microfilaremia slower than IVM and seemed to have no effect on *M perstans*. These results pave the way for further studies on the safety of MOX in loiasis, investigating higher MOX doses and patients with higher *L loa* MFDs, and possibly including female as well as male subjects.

## Supplementary Material

ofae240_Supplementary_Data

## Supplementary Data

Supplementary materials are available at *Open Forum Infectious Diseases* online. Consisting of data provided by the authors to benefit the reader, the posted materials are not copyedited and are the sole responsibility of the authors, so questions or comments should be addressed to the corresponding author.

## Notes

***Author contributions.*** C. B. C., S. D. P., M. B., and A. C. K. conceived and designed the study. G. S. W., J. T. C., J. K., and C. B. C. coordinated the study. G. S. W., T. M. L., A. E.-E., N. N.-T., A. D., L. S., G.-R. N., M. A. F. T., J. B., Y. A. B., S. M.-T., G. K. T., Y. S. N. K., F. D. M. M., and C. B. C. collected data. Y. N.-E. generated the randomization lists. H.-C. N. D. coordinated laboratory procedures. G. S. W. and T. M. L. performed the statistical analyses and wrote the first version of the manuscript. All authors critically reviewed and approved the final version of the manuscript.

***Acknowledgments.*** We are grateful to local authorities for their help and support. We thank all those who participated in the survey and those who agreed to participate in our study. We thank Medicines Development for Global Health for their support.

©World Health Organization (WHO) 2023. This is an Open Access article published under the CC BY 3.0 IGO license, which permits unrestricted use, distribution, and reproduction in any medium, provided the original work is properly cited. In any use of this article, there should be no suggestion that WHO endorses any specific organization, products, or services. The use of the WHO logo is not permitted. This notice should be preserved along with the article's original URL.

***Patient consent*.** Only individuals who signed an informed consent form were included in the study. Prior to study initiation, ethical clearance was obtained from the Cameroonian National Ethics Committee for Human Health Research (number 2021/05/1364/CE/CNERSH/SP), and an administrative authorization (number D30-1087/L/MINSANTE/SG/DROS) was obtained from the Ministry of Public Health.

***Financial support.*** This work was supported by the French National Research Agency (grant number 18-CE17-0008).

## References

[1] World Health Organization . Elimination of human onchocerciasis: progress report, 2021. Wkly Epidemiol Rec 2022:591–8.

[2] Boussinesq M, Gardon J, Gardon-Wendel N, Chippaux J-P. Clinical picture, epidemiology and outcome of *Loa*-associated serious adverse events related to mass ivermectin treatment of onchocerciasis in Cameroon. Filaria J 2003; 2:S4. 14975061 10.1186/1475-2883-2-S1-S4 PMC2147657

[3] Gardon J, Gardon-Wendel N, Demanga-Ngangue, Kamgno J, Chippaux JP, Boussinesq M. Serious reactions after mass treatment of onchocerciasis with ivermectin in an area endemic for *Loa loa* infection. Lancet 1997; 350:18–22. 9217715 10.1016/S0140-6736(96)11094-1

[4] Boussinesq M, Fobi G, Kuesel AC. Alternative treatment strategies to accelerate the elimination of onchocerciasis. Int Health 2018; 10:i40–8. 29471342 10.1093/inthealth/ihx054 PMC5881258

[5] Milton P, Hamley JID, Walker M, Basáñez M-G. Moxidectin: an oral treatment for human onchocerciasis. Expert Rev Anti Infect Ther 2020; 18:1067–81. 32715787 10.1080/14787210.2020.1792772

[6] Awadzi K, Opoku NO, Attah SK, Lazdins-Helds J, Lazdins-Helds J, Kuesel AC. A randomized, single-ascending-dose, ivermectin-controlled, double-blind study of moxidectin in *Onchocerca volvulus* infection. PLoS Negl Trop Dis 2014; 8:e2953. 24968000 10.1371/journal.pntd.0002953 PMC4072596

[7] Opoku NO, Bakajika DK, Kanza EM, et al Single dose moxidectin versus ivermectin for *Onchocerca volvulus* infection in Ghana, Liberia, and the Democratic Republic of the Congo: a randomised, controlled, double-blind phase 3 trial. Lancet 2018; 392:1207–16. 29361335 10.1016/S0140-6736(17)32844-1 PMC6172290

[8] Bakajika D, Kanza EM, Opoku NO, et al Effect of a single dose of 8 mg moxidectin or 150 μg/kg ivermectin on *O. volvulus* skin microfilariae in a randomized trial: differences between areas in the Democratic Republic of the Congo, Liberia and Ghana and impact of intensity of infection. PLoS Negl Trop Dis 2022; 16:e0010079. 35476631 10.1371/journal.pntd.0010079 PMC9084535

[9] Prichard R, Prichard R, Ménez C, Ménez C, Lespine A, Lespine A. Moxidectin and the avermectins: consanguinity but not identity. Int J Parasitol Drugs Drug Resist 2012; 2:134–53. 24533275 10.1016/j.ijpddr.2012.04.001 PMC3862425

[10] Kamgno J, Pion SD, Chesnais CB, et al A test-and-not-treat strategy for onchocerciasis in *Loa loa*–endemic areas. N Engl J Med 2017; 377:2044–52. 29116890 10.1056/NEJMoa1705026 PMC5629452

[11] Campillo JT, Hemilembolo MC, Louya F, et al Temporal variability of *Loa loa* microfilaraemia. Parasit Vectors 2023; 16:23. 36691079 10.1186/s13071-022-05612-0 PMC9869825

[12] Khwaja A . KDIGO clinical practice guidelines for acute kidney injury. Nephron Clin Pract 2012; 120:c179–84. 22890468 10.1159/000339789

[13] Jürgens S, Schulz-Key H. Effect of ivermectin on the vertical distribution of *Onchocerca volvulus* microfilariae in the skin. Trop Med Parasitol 1990; 41:165–8. 2382096

[14] Darge K, Lucius R, Monson MH, Behrendsen J, Büttner DW. Immunohistological and electron microscopic studies of microfilariae in skin and lymph nodes from onchocerciasis patients after ivermectin treatment. Trop Med Parasitol 1991; 42:361–7. 1796234

[15] Pion SD, Tchatchueng-Mbougua JB, Chesnais CB, et al Effect of a single standard dose (150–200 μg/kg) of ivermectin on *Loa loa* microfilaremia: systematic review and meta-analysis. Open Forum Infect Dis 2019; 6:ofz019. 30968052 10.1093/ofid/ofz019 PMC6449757

[16] Ducorps M, Gardon-Wendel N, Ranque S, et al Secondary effects of the treatment of hypermicrofilaremic loiasis using ivermectin [in French]. Bull Soc Pathol Exot 1995; 88:105–12. 8555762

[17] Chesnais CB, Pion SD, Boullé C, et al Individual risk of post-ivermectin serious adverse events in subjects infected with *Loa loa*. EClinicalMedicine 2020; 28:100582. 33294807 10.1016/j.eclinm.2020.100582 PMC7700892

