# Supplementary material for: Safety and Short-term Efficacy of a Single Dose of 2 mg Moxidectin in Loa loa–Infected Individuals: A Double-Blind, Randomized Ivermectin-Controlled Trial With Ascending Microfilarial Densities
Source: Open Forum Infect Dis. 2024 Apr 25;11(7):ofae240. doi: 10.1093/ofid/ofae240 (PMC11222972; doi:10.1093/ofid/ofae240)
Supplement: ofae240_Supplementary_Data [file ofae240_supplementary_data.docx]

**Safety and short-term efficacy of a single 2 mg moxidectin in *Loa loa* infected individuals: a double-blinded randomized ivermectin-controlled trial with ascending microfilarial densities**

Guy S. Wafeu (MD),^1^ Tristan M. Lepage (MD),^2,3^ Jeremy T. Campillo (PhD),^2^ Arnauld Efon-Ekangouo (MSc),^1^ Hugues-Clotaire Nana-Djeunga (PhD),^1^ Narcisse Nzune-Toche (MSc),^1^ André Domche (MSc),^1^ Laurentine Sumo (PhD),^1,4^ Guy-Roger Njitchouang (PhD),^1^ Martine Augusta Flore Tsasse (MSc),^1^ Jean Bopda (MSc),^1^ Yves Aubin Balog (MSc),^1^ Yannick Niamsi-Emalio (MSc),^1^ Stève Mbickmen-Tchana (MSc),^1^ Gervais Kamga Talla (MD),^1^ Yannick Sédrick Nguedia Kana (MD),^1^ Félicité Diane Maga Messina (MD),^1^ Sébastien D. Pion (PhD),^2^ Annette C. Kuesel (PhD),^5^ Joseph Kamgno (PhD),^1,6^ Michel Boussinesq (PhD),^2,$^ Cedric B. Chesnais (PhD).^2,$*^

*^1^ Higher Institute of Scientific and Medical Research (ISM), Yaoundé, Cameroon*

*^2^ TransVIHMI, Université de Montpellier, INSERM Unité 1175, Institut de Recherche pour le Développement (IRD), Montpellier, France*

*^3^* *Department of Infectious and Tropical Diseases, Montpellier University Hospital, Montpellier, France*

*^4^ Department of Animal Biology and Physiology, University of Ebolowa, Ebolowa, Cameroon*

*^5^ UNICEF/UNDP/World Bank/WHO Special Programme for Research and Training in Tropical Diseases (WHO/TDR), Geneva, Switzerland*

^6^ *Department of Public Health, Faculty of Medicine and Biomedical Sciences, University of Yaoundé I, Yaoundé, Cameroon.*

* Corresponding author: cedric.chesnais@ird.fr
Institut de recherche pour le développement (IRD)
911 avenue Agropolis

**Supplementary material 1. Justification of the 2 mg moxidectin dose**

To date, only two studies in animal models have been conducted to compare the effects of IVM and MOX on the MFDs of filarial species with blood-borne mf but administration by subcutaneous injection limits their informative value for MOX dose selection for studies in patients with *L loa* infection.^[[1]](#footnote-1),^^[[2]](#footnote-2)^ The most informative data comparing the short-term decrease in MFDs (and thus the safety) after a single dose of IVM and MOX as well as the long term effect on MFD are those collected during a phase 2 trial in individuals with *O volvulus* mf in the skin.^[[3]](#footnote-3)^ In this trial, 45 subjects received a single oral standard 150 µg/kg dose of IVM, and 44, 45 and 38 individuals received a single oral dose of 2 mg, 4 mg or 8 mg MOX, respectively. *O volvulus* MFDs were measured by examination of four small skin biopsies (skin snips) at D8, and at months 1, 2, 3, 6, 12 and 18. Given that post-IVM SAEs in heavily *Loa loa* infected individuals usually appear within 2-4 days after treatment,^[[4]](#footnote-4)^ we based dose selection on the change in MFD from pre-treatment to D8.^4^ These data showed that *O volvulus* MFD decrease was much faster after 4 mg or 8 mg MOX than after IVM, while the difference in MFD decrease after 2 mg MOX and IVM was not as extensive: on D8, the adjusted mean MFD change from pre-treatment after IVM, 2 mg, 4 mg or 8 mg of MOX was 0.17, 0.11, 0.08 and 0.07, respectively.^3^ Consequently, for this first trial, we chose a dose of 2 mg MOX.

**Supplementary material 2. Reasons for absence during treatment and follow-up of participants**

| **Cohorts** | **Arms** | **Reasons for absence during treatment** | **Reasons for absence during follow-up** |
| --- | --- | --- | --- |
| Cohort 1 | MOX |  | - 01 absence for travel at Day 30 |
|  | IVM | - 03 acute infection before treatment | - 01 absence for familial reasons at Day 3 - 01 absence for travel at Day 30 |
| Cohort 2 | MOX | - 02 acute infection before treatment | - 04 absence for travel at Day 30 |
|  | IVM | - 01 consent withdrawal before treatment | - 02 acute infection before treatment |
| Cohort 3 | MOX | - 03 consent withdrawal before treatment - 03 acute infection before treatment | - 02 absence for travel at Day 3 - 01 absence for familial reasons at Day 7 - 07 absence for travel at Day 30 |
|  | IVM | - 01 consent withdrawal before treatment - 01 acute infection before treatment | - 01 absence for familial reasons at Day 3 - 03 absence for travel at Day 7 - 03 absence for travel at Day 30 |

**Supplementary material 3. Description of possibly related adverse events in MOX and IVM arms, according to participants’ age.**

|  | **MOX** | | | **IVM** | | |
| --- | --- | --- | --- | --- | --- | --- |
| **Characteristic** | **18-55 *yo*,**  N = 17 | **55-70 *yo*,**  N = 19 | ***P*** | **18-55 *yo*,**  N = 21 | **55-70 *yo*,**  N = 15 | ***P*** |
| **Patient with any AE** | 11 (65%) | 17 (89%) | .114 | 14 (67%) | 12 (80%) | .468 |
| Clinical AEs^£^ | 8 (47%) | 12 (63%) | .332 | 12 (57%) | 11 (73%) | .319 |
| Laboratory AEs^£^ | 6 (35%) | 9 (47%) | .463 | 5 (24%) | 6 (40%) | .465 |
| **Grade** |  |  | .622 |  |  | .241 |
| Grade 1 | 11 (92%) | 14 (82%) |  | 10 (71%) | 5 (45%) |  |
| Grade 2 | 1 (8.3%) | 3 (18%) |  | 4 (29%) | 6 (55%) |  |
| Grade 3 & 4 AE | 0 (0%) | 0 (0%) |  | 0 (0%) | 0 (0%) |  |

^£^ Clinical AEs cases within 30 days after treatment by treatment arms; Laboratory AEs cases reported at 7 days after treatment by treatment arms.

**Supplementary material 4. Number and proportion of subjects with adverse events (AE) possibly related to study drug and severity of the AEs pre-treatment *L*. *loa* microfilarial density and treatment arm.**

|  |  | **MOX** |  |  | **IVM** |  |
| --- | --- | --- | --- | --- | --- | --- |
| **MFD category** | **1-250 mf/mL (n=20)** | **255-1000 mf/mL (n=16)** | ***P* *** | **1-250 mf/mL (n=16)** | **255-1000 mf/mL (n=20)** | ***P* *** |
| Subjects with any AE | 16 (80%) | 12 (75%) | .999 | 9 (56%) | 17 (85%) | .073 |
| Clinical AEs^£^ | 9 (45%) | 11 (69%) | .154 | 7 (44%) | 16 (80%) | .024 |
| Laboratory AEs^£^ | 11 (55%) | 4 (25%) | .070 | 5 (31%) | 6 (30%) | .999 |
| Maximum grade reached |  |  | .279 |  |  | .999 |
| Grade 1 AEs^†^ | 16 (94%) | 9 (75%) |  | 5 (56%) | 10 (62%) |  |
| Grade 2 AEs^†^ | 1 (5.9%) | 3 (25%) |  | 4 (44%) | 6 (38%) |  |
| Grade 3 & 4 AEs^†^ | 0 (0%) | 0 (0%) |  | 0 (0%) | 0 (0%) |  |

MFD=*L. loa* microfilarial density.

*Fisher's exact test, Pearson's Chi-squared test.

^†^in these lines, the proportions are calculated only on those individuals who have developed an AE.

^£^ Clinical AEs cases within 30 days after treatment by treatment arms; Laboratory AEs cases reported at 7 days after treatment by treatment arms.

**Supplementary material 5. Comparison of laboratory parameters between inclusion and D7 in MOX and IVM arms.**

|  |  | **MOX** |  |  | **IVM** |  |
| --- | --- | --- | --- | --- | --- | --- |
|  | **at inclusion** **(n=36)** | **on D7 (n=35)** | ***P*** * | **on inclusion (n=36)** | **at D7 (n=33)** | ***P*** * |
| Hemoglobin | 13.95 (13.37, 14.95) | 13.90 (13.05, 14.45) | .040 | 14.45 (13.20, 15.85) | 13.70 (12.90, 15.00) | .132 |
| Leukocytes | 7.40 (6.55, 8.88) | 7.60 (6.15, 8.90) | .476 | 7.25 (5.92, 8.40) | 7.70 (5.50, 9.60) | .221 |
| Neutrophils | 2.40 (2.07, 2.80) | 2.30 (1.85, 2.85) | .786 | 2.25 (1.90, 2.98) | 2.40 (1.80, 2.70) | .881 |
| Eosinophils | 1.50 (1.08, 2.43) | 1.50 (.80, 2.45) | .688 | 1.60 (.67, 2.02) | 1.70 (.70, 2.60) | .405 |
| Lymphocytes | 2.85 (2.40, 3.30) | 2.80 (2.35, 3.35) | .943 | 2.55 (1.97, 3.12) | 2.70 (2.10, 3.60) | .262 |
| Platelets | 212 (196, 241) | 216 (179, 256) | .694 | 194 (148, 255) | 201 (148, 234) | .543 |
| ALT | 18.1 (15.9, 22.6) | 17.5 (14.1, 22.2) | .980 | 20 (16, 26) | 19 (16, 21) | .200 |
| GGT | 21 (17, 38) | 23 (10, 43) | .235 | 34 (19, 67) | 21 (11, 40) | < .001 |
| Bilirubin | 0.93 (0.63, 1.19) | 0.88 (0.71, 1.11) | .896 | 1.08 (0.74, 1.45) | 0.93 (0.83, 1.18) | .748 |
| Creatinine | 0.99 (0.91, 1.09) | 1.03 (0.94, 1.12) | .069 | 1.02 (0.92, 1.11) | 1.06 (0.95, 1.11) | .156 |
| CRP | 7.0 (5.0, 1.0) | 7.0 (6.0, 9.5) | .948 | 6 (4, 10) | 5 (5, 13) | .583 |

Data present median values and interquartile ranges in brackets

ALT=Alanine transaminase. GGT=Gamma-glutamyltransferase. CRP=C-reactive protein.

*p values: Wilcoxon signed-rank test

**Supplementary material 6. Comparison of changes in laboratory parameters’ (% change from baseline to D7) in MOX and IVM arms.**

| **Laboratory parameters** | **MOX arm (n=35)** **^†^** | **IVM arm (n=33)** **^†^** | ***P* *** |
| --- | --- | --- | --- |
| Hemoglobin | -2 (-8, 2) | -2 (-7, 2) | .659 |
| Leukocytes | -2 (-13, 6) | 8 (-13, 23) | .167 |
| Neutrophils | 0 (-18, 19) | 0 (-19, 24) | .956 |
| Eosinophils | -4 (-18, 22) | 15 (-33, 33) | .527 |
| Lymphocytes | 0 (-17, 19) | 5 (-8, 28) | .469 |
| Platelets | -3 (-9, 11) | -3 (-19, 14) | .566 |
| ALT | -2 (-14, 13) | -8 (-21, 17) | .393 |
| GGT | -15 (-39, 30) | -31 (-74, -8) | .017 |
| Bilirubin | 1 (-13, 23) | -2 (-17, 24) | .855 |
| Creatinine | 7 (-4, 18) | 6 (-5, 17) | .652 |
| CRP | 0 (-37, 41) | 17 (-60, 300) | .629 |
| **KDIGO classification of Creatinine change** |  |  | .493 |
| Normal | 33 (94.3) | 32 (97.0) |  |
| AKI grade 1 | 2 (5.7) | 0 (0) |  |
| AKI grade 2 | 0 (0) | 1 (3.0) |  |

**^†^**Data present for laboratory parameters are median percentage of change (interquartile ranges), and n (%) for KDIGO classification.

ALT=Alanine transaminase. GGT=Gamma-glutamyltransferase. CRP=C-reactive protein, KDIGO = Kidney disease improving global outcomes.

*p values: Wilcoxon signed-rank test or Chi-squared test.

**Supplementary material 7. Number and proportion of subjects with adverse events (AE) possibly related to MOX or IVM arms, and severity of the AEs, according to the presence versus absence of *M. perstans* microfilaraemia.**

|  | MOX | | | IVM | | |
| --- | --- | --- | --- | --- | --- | --- |
| ***M. perstans* microfilaraemia** | **absent (n=28)** | **present (n=8)** | ***P* *** | **absent (n=27)** | **present (n=9)** | ***P* *** |
| Subjects with any AEs | 22 (79%) | 6 (75%) | .999 | 19 (70%) | 7 (78%) | .999 |
| Clinical AEs^£^ | 17 (61%) | 3 (38%) | .422 | 16 (59%) | 7 (78%) | .438 |
| Laboratory AEs^£^ | 10 (36%) | 5 (62%) | .236 | 9 (33%) | 2 (22%) | .690 |
| Maximum grade reached |  |  | .553 |  |  | .999 |
| Grade 1 AEs^†^ | 19 (83%) | 6 (100%) |  | 11 (58%) | 4 (67%) |  |
| Grade 2 AEs^†^ | 4 (17%) | 0 (0%) |  | 8 (42%) | 2 (33%) |  |
| Grade 3 & 4 AEs^†^ | 0 (0%) | 0 (0%) |  | 0 (0%) | 0 (0%) |  |

* Fisher's exact test, Pearson's Chi-squared test.

^†^ in these lines, the proportions are calculated only on those individuals who have developed an AE.

^£^ Clinical AEs cases within 30 days after treatment by treatment arms; Laboratory AEs cases reported at 7 days after treatment by treatment arms.

**Supplementary material 8. Mean *Mansonella perstans* microfilaremia, mean and median difference between baseline and Dx values**

| Days | MOX | | IVM | | *P* |
| --- | --- | --- | --- | --- | --- |
|  | Median [IQR] | Median relative difference (%) | Median [IQR] | Median relative difference |  |
| D0 | 57.5 [42.5 ; 1 060.0] |  | 65.0 [55.0 ; 135.0] |  |  |
| D3 | 82.5 [52.5 ; 953.8] | -17.9 [-101.2 ; 21.5] | 140.0 [90.0 ; 282.5] | -45.5 [-125.9 ; 2.0] | .694 |
| D7 | 105.0 [33.8 ; 973.8] | 3.0 [-59.4 ; 18.9] | 125.0 [62.5 ; 175.0] | 7.4 [-53.8 ; 5.0] | .536 |
| D30 | 85.0 [60.0 ; 435.0] | -4.8 [-74.5 ; 46.0] | 170.0 [67.5 ; 260.0] | -27.3 [-92.6 ; 7.1] | .639 |

*p value of Wilcoxon rank-sum test, comparing Median relative difference. This table only include participants infected with Mansonella perstans (n = 17).*

`

**Supplementary material 9. Mean microfilaremia, mean and median difference between baseline and Dx values *(Per protocol analysis)***

| Days | MOX (n = 24) | | IVM (n = 31) | | *P* |
| --- | --- | --- | --- | --- | --- |
|  | Median [IQR] | Median relative difference (%) | Median [IQR] | Median relative difference |  |
| D0 | 110.0 [31.3 ; 396.3] |  | 275.0 [92.5 ; 460.0] |  |  |
| D3 | 97.5 [28.8 ; 191.3] | 47.3 [-115.2 ; 69.3] | 80.0 [27.5 ; 180.5] | 69.5 [41.1 ; 85.0] | .012 |
| D7 | 65.0 [13.8 ; 171.3] | 55.2 [-37.5 ; 78.2] | 80.0 [12.5 ; 187.5] | 76.4 [56.0 ; 88.9] | .049 |
| D30 | 72.5 [18.8 ; 217.5] | 48.1 [-12.7 ; 81.7] | 65.0 [10.0 ; 220.0] | 77.2 [33.3 ; 97.8] | .062 |

*p value of Wilcoxon rank-sum test, comparing Median relative difference*

**Supplementary material 10. Proportion of participants with 40%, 80% and 100% reduction in their microfilaremia *(Per protocol analysis)***

| Days | 40% Decrease | | | | | 80% decrease | | | | | Microfilaria clearance | | | | |
| --- | --- | --- | --- | --- | --- | --- | --- | --- | --- | --- | --- | --- | --- | --- | --- |
|  | MOX (n = 24) | | IVM (n = 31) | | ***P*** | MOX (n = 24) | | IVM (n = 31) | | ***P*** | MOX (n = 24) | | IVM (n = 31) | | ***P*** |
|  | Yes | No | Yes | No |  | Yes | No | Yes | No |  | Yes | No | Yes | No |  |
| D3 | 14 (58.3) | 10 (41.7) | 24 (77.4) | 7 (22.6) | .129 | 3 (12.5) | 21 (87.5) | 10 (32.3) | 21 (67.7) | .087 | 1 (4.2) | 23 (98.8) | 3 (9.7) | 28 (9.3) | .624 |
| D7 | 14 (58.3) | 10 (41.7) | 26 (83.9) | 5 (16.1) | .035 | 5 (2.8) | 19 (79.2) | 12 (38.7) | 19 (61.3) | .155 | 3 (12.5) | 21 (87.5) | 5 (16.1) | 26 (83.9) | .999 |
| D30 | 13 (54.2) | 23 (74.2) | 11 (45.8) | 8 (25.8) | .121 | 6 (25) | 18 (75) | 15 (48.4) | 16 (51.6) | .077 | 4 (16.7) | 20 (83.3) | 7 (22.6) | 24 (77.4) | .738 |

**Supplementary material 11**. Number of adverse events possibly related to treatment (AEs), number of subjects having developed such AEs, and interval of time between treatment and onset of AEs in the two treatment arms ***(Per protocol analysis)***

| **Adverse events** | **MOX** **(n=24)^£^** | **IVM (n=31) ^£^** | ***P*** |
| --- | --- | --- | --- |
| Median onset, days (IQR) | 3.0 (1.0, 7.0) | 2.0 (1.0, 6.0) | .833* |
| Mean onset, days (IQR) | 5.2 (1.0, 7.0) | 4.6 (1.0, 6.0) | .833* |
| Number of AEs | 37 | 60 |  |
| Clinical AEs | 22 | 40 |  |
| ≤ 7 days | 18 | 33 |  |
| > 7 days^¥^ | 4 | 7 |  |
| Laboratory AEs at D7 | 15 | 20 |  |
| Number of subjects with AEs (%) |  |  |  |
| Any AEs | 21 (87.5%) | 23 (74.2%) | .314*** |
| Clinical AEs | 15 (62.5%) | 20 (64.5%) | .877** |
| ≤ 7 days | 12 (50.0%) | 18 (58.1%) | .551** |
| > 7 days^¥^ | 5 (20.8%) | 4 (12.9%) | .482*** |
| Laboratory AEs | 10 (41.7%) | 10 (32.3%) | .472** |
| Maximum grade reached |  |  |  |
| Grade 1 AE^†^ | 18 (85.7%) | 11 (47.8%) | .008*** |
| Grade 2 AE^†^ | 3 (14.3%) | 12 (52.2%) |  |
| Grade 3 & 4 AE | 0 (0%) | 0 (0%) |  |

MOX=moxidectin. IVM=ivermectin. IQR=interquartile range;

^£^ n: Number of participants included in the arm.

* p values for Wilcoxon rank-sum test.

** p values for Pearson's Chi-squared test.

*** p values for Fisher’s Exact test.

^¥^ Clinical AEs were reported up to 30 days.

^†^ In these lines, the proportions are calculated only on those individuals who have developed an AE

1. Schares G, Hofmann B, Zahner H. Antifilarial activity of macrocyclic lactones: comparative studies with ivermectin, doramectin, milbemycin A4 oxime, and moxidectin in *Litomosoides carinii*, *Acanthocheilonema viteae*, *Brugia malayi*, and *B. pahangi* infection of *Mastomys coucha*. *Trop Med Parasitol Off Organ Dtsch Tropenmedizinische Ges Dtsch Ges Tech Zusammenarbeit GTZ* 1994; **45**: 97–106. [↑](#footnote-ref-1)
2. Breton B, Diagne M, Wanji S, *et al.* Ivermectin and moxidectin in two filarial systems: resistance of *Monanema martini*; inhibition of *Litomosoides sigmodontis* insemination. *Parassitologia* 1997; **39**: 19–28. [↑](#footnote-ref-2)
3. Awadzi K, Opoku NO, Attah SK, Lazdins-Helds J, Kuesel AC. A randomized, single-ascending-dose, ivermectin-controlled, double-blind study of moxidectin in *Onchocerca volvulus* infection. *PLoS Negl Trop Dis* 2014; **8**: e2953. [↑](#footnote-ref-3)
4. Boussinesq M, Gardon J, Gardon-Wendel N, Chippaux J-P. Clinical picture, epidemiology and outcome of *Loa*-associated serious adverse events related to mass ivermectin treatment of onchocerciasis in Cameroon. *Filaria J* 2003; **2**: S4. [↑](#footnote-ref-4)
